# From motor performance to participation: a quantitative descriptive study in adults with autosomal recessive spastic ataxia of Charlevoix-Saguenay

**DOI:** 10.1186/s13023-018-0898-z

**Published:** 2018-09-19

**Authors:** Cynthia Gagnon, Bernard Brais, Isabelle Lessard, Caroline Lavoie, Isabelle Côté, Jean Mathieu

**Affiliations:** 1 Centre de recherche Charles-Le Moyne – Saguenay–Lac-St-Jean sur les innovations en santé, Université de Sherbrooke, Québec, Canada; 2 Faculté de médecine et des sciences de la santé, Université de Sherbrooke, Québec, Canada; 3 Groupe de recherche interdisciplinaire sur les maladies neuromusculaires (GRIMN), Centre intégré universitaire de santé et de services sociaux du Saguenay-Lac-St-Jean, 2230 de l’Hôpital, C.P. 1200, Jonquière (Québec), G7X 7X2 Canada; 4 Montreal Neurological Institute, McGill University, Quebec, Canada

**Keywords:** ARSACS, Recessive ataxia, Quantitative motor performance, Functional capacity, Disease severity, Natural history, Mobility limitation, Activities of daily living

## Abstract

**Background:**

Autosomal Recessive Spastic Ataxia of Charlevoix-Saguenay (ARSACS) is a recessive neurological disorder with cerebellar, pyramidal and neuropathic features. Natural history data are urgently needed to increase trial readiness. This study aimed to describe the clinical phenotype including dexterity, coordination, strength, mobility, balance, disease severity, participation, and quality of life observed in adults with ARSACS homozygous for the c.8844delT mutation.

**Methods:**

Cross-sectional study with comparisons between disease stages and with reference values. Outcome measures included Standardized Finger-to-Nose Test, Grip/pinch strength, LEMOCOT, Six-Minute Walk Test, 10-Meter Walk Test, Berg Balance Scale, Spastic Paraplegia Rating Scale, Scale for the Assessment and Rating of Ataxia, LIFE-H, and SF-12.

**Results:**

Twenty-eight participants were recruited with a mean age of 38.1 years. The majority presented with lower limb coordination and fine dexterity scores below three standard deviations compare to reference values, scored under predicted values for mobility measures and were at increased risk of fall. Participants at an earlier disease stage performed better than the others, but individual variability was observed.

**Conclusions:**

Results showed overall impaired motor performances and, even in a genetically homogeneous ARSACS population, an individual variability within disease stages. This study lays the foundation for a longitudinal study using quantified measurements.

## Background

Autosomal recessive spastic ataxia of Charlevoix-Saguenay (ARSACS) is an autosomal recessive disorder more prevalent in the French-Canadian population [1] but with cohorts reported worldwide [2, 3]. International prevalence of ARSACS is not well known, but the carrier rate was estimated at 1/22 inhabitants in the Saguenay-Lac-St-Jean region (Quebec, Canada), and the incidence at birth at 1/1932 liveborn infants [4]. ARSACS is caused by mutations in the *SACS* gene [5] which is located on chromosome 13q12 [6]. Most of the French-Canadian cases (92.6%) are homozygous for the c.8844delT mutation and do not produce any sacsin protein [7]. Over 150 other mutations have been identified internationally [7]. These mutations lead to different level of sacsin protein expression, that may contribute to the differences observed in phenotypes [8].

Based on clinical observations and review of medical records, ARSACS clinical phenotype of French Canadian cases has been previously described. It consists of an early childhood onset of the disease with youngsters generally showing unsteadiness at gait initiation [6, 9]. Walking is delayed in most cases to around 18 months of age and walking difficulties is often the symptom leading to the first consultation [10]. The disease progression becomes most obvious in the late teens or early twenties [9]. Individuals will lose walking around their forties [9] but may experience severe walking limitations as soon as their early adulthood. Mean age of becoming constant wheelchair user is 41 years with a large range of 17 to 58 years [9], illustrating the great variability in the clinical spectrum even among a genetically homogeneous cohort. However, the documentation of the clinical portrait of ARSACS using quantified testing is scarce, and only one study has documented the impact of ARSACS on functional autonomy and participation [11]. So it is highly complex for clinicians to give prognostic in regard to disease severity and functional impacts, or anticipate the future steps and interventions needed for their patients since data do not exist in the literature. In addition, the increasing knowledge of ARSACS’ pathophysiology and the availability of a good mice transgenic model [12] increase the likelihood that clinical trials will be launched in a foreseeable future. But natural history studies using quantified measures are essential in order to design robust clinical trial protocols. As stated by the U.S. Food and Drug Administration, natural history studies are essential to provide the scientific foundation to build drug development programs, which require a deep understanding of the disease. The more these data are available early, the more it is informative to design efficacy trials [13].

This cross-sectional study aimed to: 1) Document motor performance in a genetically homogeneous cohort of adults with ARSACS in terms of dexterity, coordination, strength, mobility, and balance and overall disease severity; 2) Explore other systems involvement; 3) Document participation and health-related quality of life; and 4) Compare patients’ performances between different disease stages, age groups and with reference values.

## Method

### Subjects

Participants were recruited among a subset of 175 patients with ARSACS followed at the Neuromuscular Clinic of the *Centre Intégré Universitaire de Santé et de Services Sociaux du Saguenay–Lac-St-Jean* (Quebec, Canada), as described in Lessard et al. [14]. Briefly, participants needed to be 18 years old or older with a diagnosis of ARSACS confirmed by DNA analysis, to be homozygous for the common c.8844delT mutation, and not be affected by other pathologies causing functional limitations. The study was approved by the Ethics Review Board of the *Centre Intégré Universitaire de Santé et de Services Sociaux du Saguenay-Lac-St-Jean* (Quebec, Canada) and informed consent was obtained from all participants.

### Data collection

Participants were seen over three half-day sessions within a 2-week interval. Each session was balanced in term of difficulty and time taken to administrate tests in order to avoid fatigue of participants. A questionnaire was administered for age, sex, mobility level, and use of walking aids. Disease stage were defined based on the Scale for the Assessment and Rating of Ataxia (SARA) development study [15]): 1) No walking difficulty; 2) First walking difficulty but no use of walking aid; 3) Walking with aid or support; and 4) Wheelchair user. In addition, we ensure that all participants understood the task to perform prior to administration of tests and questionnaires (verbal ascertainment or demonstration of the task).

### Outcome measures

#### Upper limb functions (dexterity, coordination, strength)

The fine finger dexterity was measured using the Purdue Pegboard Test [16] (PPT) and Nine-Hole Peg Test [17] (NHPT). For the PPT, the number of pegs placed on the board during a 30-s period was counted (2 trials). The NHPT consist of placing and removing nine pegs from holes on a board as quickly as possible and the time to complete the task in seconds is recorded (2 trials). To measure upper extremity motor coordination, the Standardized Finger-Nose Test [18] (SFNT) was used. With their index finger participants move horizontally from their nose to a target placed 45 cm away (2 trials) as quickly as possible in a 20-s period. Intra- and interrater reliability of the NHPT and SFNT are excellent (ICC = 0.90–0.98) and their construct validity has been recently demonstrated in ARSACS [19]. Grip strength was measured using a Jamar dynamometer (Asimow Engineering Co., Los Angeles, CA) and lateral pinch between the thumb and index finger was measured using a pinch gauge (Baseline Pinch Gauge, Fabrication Enterprises Inc., Irvington, NY) (3 trials).

#### Lower limb coordination

Coordination of lower limbs was assessed using the Lower Extremity Motor Coordination Test (LEMOCOT). Sitting down, participants alternatively touch two targets placed 30 cm from each other as fast as possible for 20 s (2 trials) [14, 20]. The intra- and interrater reliability of the LEMOCOT are excellent in ARSACS (ICC = 0.92–0.97), as well as its construct validity among this cohort of participants [14].

#### Mobility and balance

The Six-Minute Walk Test (6MWT) was used as a measure of walking endurance. The maximal distance walked along a 30-m linear corridor over a 6-min period was recorded (1 trial) [21]. Short distance walking speed was assessed with the 10-Meter Walk Test (10mWT) at comfortable speed (2 trials). This test measures the time required to cover a 10-m distance. Both tests (10mWT and 6MWT) have excellent interrater reliability (ICC = 0.97–0.99) and construct validity was confirmed in the ARSACS population in a recent study [22]. The Berg Balance Scale [23] (BBS) was used to assess balance and fall risk. It includes 14 items graded from 0 to 4, for a maximum score of 56 (higher values indicate better performance). Its construct validity was recently demonstrated in ARSACS [22].

#### Disease severity scales

Spastic Paraplegia Rating Scale [24] (SPRS) was used to determine the severity of spastic signs. It includes 13 items graded from 0 to 4, for a maximum score of 52 (higher score indicates more severe impairment). Cerebellar ataxia was quantified using the SARA [15], which includes eight items for a total score varying from 0 (no ataxia) to 40 (most severe ataxia).

#### Other systems involvement

An exploration of symptoms in terms of presence and severity was done by a trained physiatrist resident that interviewed participants and reviewed their medical file from the neuromuscular clinic. Symptoms were defined as: 1) Dysphagia – chocking when eating or drinking; 2) Spasms – Modified Penn Scale [25] with category grouping secondary due to sample size (0 = no spasm, 1 = induced spasms, 2 = regrouped levels 2–4 in a spontaneous spasms category); 3) Bladder problems – scale including none, presence of urgency or pollakiuria with or without treatment and incontinence controlled or uncontrolled with treatment.

#### Participation and health-related quality of life

The Assessment of Life Habits Questionnaire [26] (LIFE-H) was used to assess participation. It includes a total of 77 items and the total score is reported on nine (nine representing no difficulty to perform the activity). The Barthel Index [27], a 10-item tool, measures independence level in activity of daily living and mobility. The maximum score is 100 and represents total independence. Health-related quality of life was assessed using the 12-item Short-Form Health Survey [28] (SF-12), that generates two composite scores: the Mental Component Summary (MCS) and the Physical Component Summary (PCS). The maximum score for each component is 100. In addition, a demographic questionnaire was completed.

### Statistical analysis

Data are expressed as mean ± standard deviation (SD) for continuous variables and as frequency and percentage for categorical variables. When more than one trial was made, the mean was used for analyses. Participants were compared to non-participants using a Mann-Whitney U Test for the age and a Chi-Square test for independence for the sex. Performance between disease stages were compared using a Kruskal-Wallis test due to a number of participants lower than 30. A *p* value < 0.05 was considered significant. Total scores of all outcome measures were correlated with the participant’s age using the Spearman *ρ* coefficient to demonstrate and quantify the degenerative aspect of the disease. Only results from the dominant side are presented. To compare with reference value, results from LEMOCOT [29], NHPT [30], grip strength [31] and pinch strength [31] were transformed as z-scores (i.e. the number of standard deviation the participant’s score is from the reference value). Number and percentage of participants for each z-scores categories (1.5 to − 1.5 SD, − 1.51 to − 3.0 SD, − 3.01 SD and above) are presented. Mobility scores were compared to reference values from a meta-analysis for the 10mWT [32] and predicted values according to regression equation (age and sex) for the 6MWT [33]. For the BBS, a cut-off score of < 45 was used to determine which individuals were at increased risk of fall [34, 35]. Data were analysed using IBM SPSS Statistics for Windows, Version 24.0 (Armonk, NY: IBM Corp).

## Results

### Characteristics of the cohort

All characteristics are presented in Table 1. From the 175 ARSACS cases followed at the neuromuscular clinic, 59 persons met inclusion criteria among which 15 refused to participate, 14 were not contacted when a sufficient number of participants were recruited in their age groups, and two persons dropped-out. The 28 participants included in the study have a mean age of 38.1 years and 57.1% were men. Ten participants are constant-wheelchair users. The 31 eligible cases that were not recruited were similar to the participants in terms of age (mean = 34 ± 13, *p* = 0.245) and sex (48.4% men, *p* = 0.604).

**Table 1:** Characteristics of the study population (*n* = 28)

Characteristic	Total group	No walking difficulty *n* = 7	Walking aid *n* = 11	Wheelchair *n* = 10
Age, (y)				
Mean (SD)	38.1 (12.6)	26.0 (5.7)	34.7 (9.1)	50.3 (8.5)
Range	18–59	18–33	21–50	32–59
Sex, *n* (%)				
Men	16 (57.1)	4 (57.1)	5 (45.5)	7 (70.0)
Women	12 (42.9)	3 (42.9)	6 (54.5)	3 (30.0)
Age groups, *n (%)*				
< 40 years	16 (57.1)	7 (100.0)	8 (72.7)	1 (10.0)
≥ 40 years	12 (42.9)	0	3 (27.3)	9 (90.0)
Age of indoor wheelchair use (y) (n = 10)				
Mean (SD)	38.9 (7.7)	–	–	38.9 (7.7)
Range	30–49	–	–	30–49

### Participants’ performance

Results obtained for the total sample and for each disease stage are presented in Table 2. A significant difference between the three groups was found for 11 out of 15 variables; only grip and pinch strength, and health-related quality of life as measured by SF-12v2 (MCS-PCS) did not show any significant differences. Of note that two subjects in the Wheelchair disease stage were able to perform the 10mWT and one performed the 6MWT.

**Table 2:** Performance comparison in clinical variables between ARSACS patients at different disease stages

	Total	No walking difficulty	Walking aid	Wheelchair	*p*-value^**^
	*n* = 28	*n* = 7	*n* = 11	*n* = 10	
Upper limb functions (dexterity, coordination and strength)					
PPT (no. of pegs)	5.2 (2.4)^a^	7.9 (0.90)	5.4 (1.6)	2.8 (1.7)	***< 0.001***
min-max	0.5–9.5	6.5–9.5	3.0–8.5	0.5–6.5	
NHPT (seconds)	58.2 (37.7)	33.8 (3.5)	45.0 (8.8)	89.6 (49.0)	***< 0.001***
min-max	28.6–211.2	28.6–37.5	35.1–61.0	35.9–211.2	
SFNT (no. of targets)	11.5 (3.7)	14.4 (4.2)	12.4 (1.7)	8.4 (3.0)	***0.002***
min-max	5.5–23.0	10.0–23.0	10.5–15.0	5.5–15.5	
Grip strength (kg)	28.9 (9.8)	30.0 (11.9)	27.8 (10.1)	29.2 (9.0)	0.736
min-max	11.3–52.3	13.3–44.0	16.7–52.3	11.3–38.3	
Pinch strength (kg)	6.8 (1.9)	7.6 (1.6)	6.1 (1.5)	7.0 (2.3)	0.294
min-max	3.7–10.5	4.5–9.3	4.1–8.8	3.7–10.5	
Lower limb functions (coordination)					
LEMOCOT (no. of targets)	17.0 (10.1)	27.7 (3.6)	19.3 (5.8)	6.9 (7.3)	***< 0.001***
min-max	0–34.5	22.0–32.5	13.5–34.5	0–21.5	
Mobility and balance					
6MWT^b^ (meters)	235.3 (116.3)	355.0 (78.8)	177.4 (53.9)	35.0^c^	***0.002***
min-max	35.0–459.5	256.0–459.5	99.0–270.0	35.0	
10mWT^b^ (speed, m/s)	0.87 (0.44)	1.33 (0.21)	0.71 (0.26)	0.15 (0.02)^c^	***0.001***
min-max	0.14–1.6	1.1–1.6	0.33–1.2	0.14–0.17	
BBS	22.1 (19.1)	47.3 (5.8)	24.0 (10.6)	2.5 (4.2)	***< 0.001***
min-max	0–56	42–56	11–42	0–13	
Disease severity					
SPRS	24.3 (11.2)	11.1 (4.3)	21.8 (4.1)	36.1 (6.9)	***< 0.001***
min-max	3.0–50.0	3.0–16.0	16.0–28.0	26.0–50.0	
SARA	20.6 (8.9)	10.3 (3.0)	18.0 (2.3)	30.7 (4.5)	***< 0.001***
min-max	6.0–36.0	6.0–13.5	13.0–22.0	23.0–36.0	
Participation and health-related quality of life					
LIFE-H	7.8 (1.2)	9.3 (0.57)	7.7 (0.50)	6.9 (1.4)	***0.001***
min-max	4.0–9.7	8.3–9.7	6.9–8.6	4.0–8.9	
Barthel Index	84.3 (22.1)	99.3 (1.9)	94.1 (7.0)	63.0 (24.9)	***< 0.001***
min-max	25–100	95–100	80–100	25–95	
SF-12v2 (MCS)	54.9 (12.8)	51.8 (13.3)	59.9 (9.4)	51.5 (15.0)	0.141
min-max	13.1–72.5	22.8–61.5	39.8–72.5	13.1–66.2	
SF-12v2 (PCS)	41.4 (5.8)	41.4 (6.6)	39.4 (5.5)	43.6 (5.3)	0.333
min-max	27.0–51.5	32.3–49.9	27.0–46.7	38.0–51.5	

**Comparison between < 40 years and ≥ 40 years using a Krsukal-Wallis Test; results in bold are significant (*p*-value < 0.05)

^a^Results are presented as Mean (standard deviation)

^b^19 participants were able to performed the 6MWT and 20 participants the 10mWT in the total sample

^c^One participant out of 10 have performed the 6MWT and two participants performed the 10mWT in the Wheelchair disease stage

**Fig. 1:**
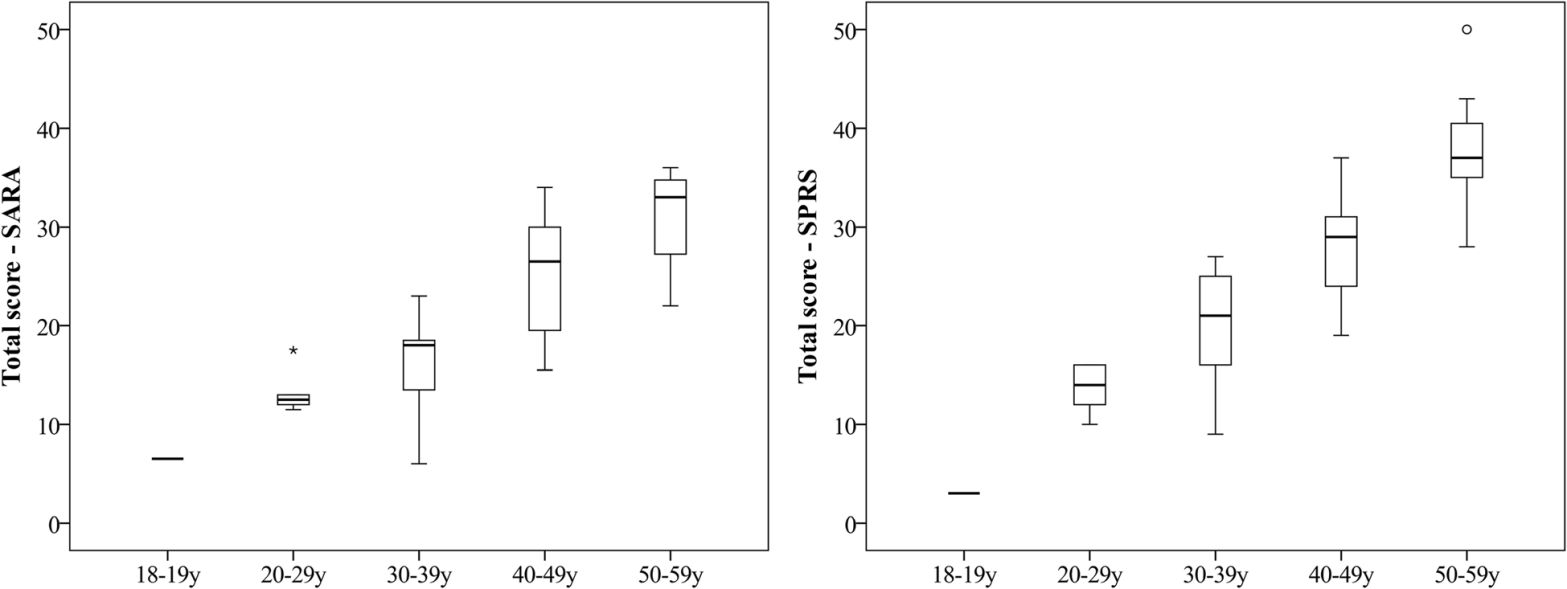
Comparison of the severity of ataxia as measured by the *Scale for the Assessment and Rating of Ataxia* (SARA) and spasticity measured by the S*pastic Paraplegia Rating Scale* (SPRS) scores between age groups

The correlation between all tests’ score and participant’s age was explored to transversely illustrate the progression of the disease. Except for strength and quality of life, all correlations were significant (ρ = ± 0.64–0.87). Although this high correlation is well illustrated in the Fig. 1 for the disease severity as measured by the SARA and the SPRS, this figure also illustrates the variability within a group of participants at the same age. For example, SARA scores vary from 6 to 23 for participants aged between 30 and 39-year old and SPRS scores vary from 9 to 27 for the same age group.

### Comparisons with reference values

Comparison of results with reference values are presented in Table 3. In addition, only 4 participants (20.0%) were within the predicted value for the 10mWT, and all of them were under 40 years old. For the 6MWT, participants obtained between 3.0 and 66.0% of expected value. Results also show that 24 participants (85%) obtained a score below 45 at the BBS, indicating that they are at greater risk of falling.

**Table 3:** Comparison of lower limb coordination, upper limb strength and dexterity with reference values

Outcome measures	Age groups	Standard deviation from reference value		
		1.5 to − 1.5	−1.51 to − 3.0	−3.01 and below
LEMOCOT	< 40 years	1 (6.3)^a^	4 (25.0)	11 (68.8)
	≥40 years	0 (0)	2 (16.7)	10 (83.3)
Grip strength	< 40 years	4 (25.0)	7 (43.8)	5 (31.2)
	≥40 years	1 (8.3)	10 (83.3)	1 (8.3)
Pinch strength	< 40 years	10 (62.5)	5 (31.2)	1 (6.2)
	≥40 years	4 (33.3)	7 (58.3)	1 (8.3)
NHPT	< 40 years	0 (0)	0 (0)	16 (100.0)
	≥40 years	0 (0)	0 (0)	12 (100.0)

^a^All results are presented as Number of participants (%)

### Other systems involvement

Regarding other related symptoms, dysphagia was reported by 75.0% of our participants with all participants over 40 years old describing difficulties with certain foods. Also, 57.1% reported spontaneous spasms. Finally, 25.0% reported urge or pollakiuria with or without treatment and 42.9% incontinence controlled or uncontrolled with treatment.

## Discussion

This is the first study to document motor performance as well as overall disease severity using quantitative assessment among a cohort of ARSACS patients homozygous for the c.8844delT mutation. This is the first step in the documentation of the natural history of the disease, which is an essential step in drug development programs and their eventually trial for efficacy [13]. These results may also serve as comparative data for clinicians to anticipate disease progression of their patients.

This study illustrates the high level of variability within disease stage in regard to clinical presentation and disease severity. ARSACS is a progressive disease where overall severity increase with age, but results show important differences in performance level between individuals within a disease stage or age group. Despite its genetic homogeneity, a great variability in ataxia and spasticity severity as measured respectively by SARA and SPRS was observed across age groups. However, since participants’ number in each age group is small (5 to 10 participants/group) the extent of disease severity variability still needs to be further studied with larger cohorts. The age to become constant wheelchair user also illustrates the large clinical variability (mean 38.9; SD ±7.7), though close to the previously reported 41 years-old [9], with a wide range from 30 to 49 years, with even some not yet wheelchair-bound by age 50.

When comparing our results with those obtained in other ARSACS populations, disease severity as assessed by the SARA is similar to the results obtained by Vermeer et al. in 2008 in a cohort of 16 cases within the same age range (SARA mean score = 22.2; ranging from 14 to 28) [36]. However, disease severity of our cohort is slightly higher than ARSACS cases (*n* = 8) assessed by Synofzik et al. (mean age = 35.4 ± 6.6, SARA mean score = 16.1 ± 7.0) [37].

There is yet no longitudinal study in ARSACS but our study, to some extent, captures some aspects of progression by correlating the performance with participants’ age. Gagnon et al. [11] have previously observed this progression over time in upper limb tasks. However, the performance of the younger group is already below reference values for most upper limb tasks underlining early impairment onset. For grip and pinch strength, no significant correlations were seen with age, a result similar to Gagnon et al. [11]. However, since the progressive intrinsic hand muscles weakness is usually observed in the clinic at an early stage, it is possible that the relatively small sample size may have lead to a type II error, meaning that the existing difference was not detected.

The poor performance of the younger group as compared to reference value also supports lower limbs functions, balance and mobility early impairment in ARSACS. As indicated by the BBS score, 75% of younger and 100% of older participants are at high risk of falling (BBS score < 45) while walking, transferring or simply standing up without support. A recent study in Friedreich ataxia has shown a mean BBS score of 48 ± 1.3 for a group of seven participants aged from 21 to 43 years [38], well illustrating the high level of balance impairment of our participants, where the younger participants obtained a mean score of 34.6.

In regard to other symptoms less frequently associated with ARSACS in the literature, three symptoms have been pointed out. Dysphagia, which has been previously reported in 30% [36] and 35.7% [1] of patients with ARSACS, was reported in 21 participants (75%) in this study. Vesical problems were also previously reported in ARSACS [1, 36], with an incidence of about 50% of cases presenting with urine urgency and incontinence, compared to 75% in our cohort. This problem seemed to be more prevalent among older participants. Spasms are associated with presence of spasticity and upper motoneurons lesions but this symptom has never been reported in previous study. These results highlight the importance to systematically ask about dysphagia, vesical problems and spasms using standardized assessments in the follow-up because of the possible impact on quality of life and the availability of treatment.

If we look at the participation level of this ARSACS cohort, participants in the Wheelchair and Walking aid disease stages reported a lower level of participation as measured by the LIFE-H, with a mean score of 6.9 and 7.7 respectively, compared to a LIFE-H score of 9 which mean an absence of participation restriction [39]. The lower performance of participants on most upper and lower functions, mobility, and balance outcome measures may explain the lower level of social participation in these two more severe disease stages. However, decrease of physical performance does not influence Health-related quality of life. Results seem to be comparable with those of 7069 US healthy people (mean age: 50.7 years), who obtained a mean of 50 ± 10 for both SF-12 PCS and MCS composite scores [40]. However, cautions are needed in regard to these results as although often consider a gold standard, the use of SF-12v2 in a slowly progressive disease is questionable in regards to the short time lapse reference of 4 weeks used throughout the questionnaire where the condition is most likely to have been stable.

Some limitations of this study can be highlighted. Among these, we can note the overall small number of participants in the study, and particularly the small number of older participants who were able to perform the walking tests (6MWT and 10mWT). This clearly limits the power of the statistical analyses to detect a difference, although most of the comparisons were statistically significant. The other limitation is the homogeneity of our sample, which may limit to some extent the generalizability of the study in other ARSACS populations where the specific mutation causes only partial protein production in opposition to complete absence of protein production in our population [7]. Finally, some additional steps need to be accomplished to have a global portrait of the ARSACS population: 1) muscle strength impairment is not well documented, but a protocol must be first developed since presence of lower limbs co-contractions prevent valid assessment of muscle strength, and 2) the disease presentation in the paediatric population should be documented in regard to prognosis, care recommendations and trial readiness.

## Conclusions

Altogether, results showed overall impaired motor performances, but this study is the first to clearly demonstrate the high level of clinical variability in ARSACS patients homozygous for the same mutation at different ages. Despite the early mild impairments, the underperformance of the younger participants compared to the reference values underline the insidious onset of this disease that is increasingly being diagnosed by next generation sequencing worldwide. This study represents a unique source of quantified data about ARSACS adult population for clinicians and lays the foundation for future clinical trials.

## Abbreviations

10mWT: 10-Meter Walk Test
6MWT: Six-Minute Walk Test
ARSACS: Autosomal recessive spastic ataxia of Charlevoix-Saguenay
BBS: Berg Balance Scale
LEMOCOT: Lower Extremity Motor Coordination Test
LIFE-H: Assessment of Life Habits Questionnaire
MCS: Mental Component Summary
NHPT: Nine-Hole Peg Test
PCS: Physical Component Summary
PPT: Purdue Pegboard Test
SARA: Scale for the Assessment and Rating of Ataxia
SD: Standard deviation
SF-12: 12-item Short-Form Health Survey
SFNT: Standardized Finger-Nose Test
SPRS: Spastic Paraplegia Rating Scale
